# Rifaximin Protects against Malathion-Induced Rat Testicular Toxicity: A Possible Clue on Modulating Gut Microbiome and Inhibition of Oxidative Stress by Mitophagy

**DOI:** 10.3390/molecules27134069

**Published:** 2022-06-24

**Authors:** Nesreen Nabil Omar, Rasha A. Mosbah, Wedad S. Sarawi, Marwa Medhet Rashed, Amira M. Badr

**Affiliations:** 1 Department of Biochemistry, Faculty of Pharmacy, Modern University for Technology and Information, Cairo 11585, Egypt; 2 Infection Control Unit, Zagazig University Hospital, Zagazig University, El Sharkia 44519, Egypt; rashamosbah@hotmail.com; 3 Department of Pharmacology and Toxicology, King Saud University, Riyadh 11362, Saudi Arabia; wsarawi@ksu.edu.sa (W.S.S.); or amibadr@ksu.edu.sa (A.M.B.); 4 National Center for Social & Criminological Research, Expert, Crime Investigation Department, Giza 3755153, Egypt; mannna21@hotmail.com; 5 Faculty of Pharmacy, Ain Shams University, Cairo 11566, Egypt

**Keywords:** rifaximin, testicular toxicity, malathion, gut microbiome, oxidative stress, mitophagy

## Abstract

Testicular dysfunction is caused by chronic exposure to environmental pollution, such as malathion, which causes oxidative stress, promoting cell damage. Autophagy is a key cellular process for eliminating malfunctioning organelles, such as the mitochondria (mitophagy), an eminent source of reactive oxygen species (ROS). Autophagy is crucial for protection against testicular damage. Rifaximin (RFX) is a non-absorbable antibiotic that can reshape the gut microbiome, making it effective in different gastrointestinal disorders. Interestingly, the gut microbiome produces short chain fatty acids (SCFAs) in the circulation, which act as signal molecules to regulate the autophagy. In this study, we investigated the regulatory effects of RFX on gut microbiota and its circulating metabolites SCFA and linked them with the autophagy in testicular tissues in response to malathion administration. Moreover, we divided the groups of rats that used malathion and RFX into a two-week group to investigate the mitophagy process and a four-week group to study mitochondriogenesis. The current study revealed that after two weeks of cotreatment with RFX, apoptosis was inhibited, oxidative stress was improved, and autophagy was induced. More specifically, PINK1 was overexpressed, identifying mitophagy activation. After four weeks of cotreatment with RFX, there was an increase in acetate and propionate-producing microflora, as well as the circulating levels of SCFAs. In accordance with this, the expression of PGC-1α, a downstream to SCFAs action on their receptors, was activated. PGC-1α is an upstream activator of mitophagy and mitochondriogenesis. In this sense, the protein expression of TFAM, which regulates the mitochondrial genome, was upregulated along with a significant decrease in apoptosis and oxidative stress. Conclusion: we found that RFX has a positive regulatory effect on mitophagy and mitochondria biogenesis, which could explain the novel role played by RFX in preventing the adverse effects of malathion on testicular tissue.

## 1. Introduction

Testicular dysfunction is a chronic disorder of unclear etiology, characterized by the destruction of the germ cells of seminiferous tubules in addition to other supplementary cells, such as Sertoli cells and Leydig cells [1]. Environmental factors, especially pesticides, confer toxic effects on testicular tissue, which explains the increased rate of reproductive toxicity during life [2]. Malathion is an organophosphorus (OP) pesticide and has been found to interrupt the sperm quality and reduce reproductive hormones production through triggering a stream of oxidative stress [3].

Malathion generates reactive oxygen species (ROS) and decreases the activity of antioxidant enzymes, resulting in misfolded proteins and damaged organelles [4]. Moreover, Malathion disrupts autophagy [5], a quality control cellular machinery that removes damaged organelles and protein [6]. In fact, autophagy is crucial for eliminating malfunctioning mitochondria, an eminent source of ROS and cell death factors [7]. Importantly, autophagy has been shown to enhance acrosome biogenesis [8] and sperm cell differentiation during the process of spermatogenesis [9]. Previously, autophagy supported homeostasis of organisms and was found crucial for protection against testicular damage induced by hyperglycemia [10] and hypoxia [11]. Recently, spermatogenesis process was improved by autophagy, which cleared the damaged mitochondria in a process called mitophagy, therefore maintained a healthy mitochondrial network [12]. 

Recently, the gut–testis axis has been introduced as the origin of impaired spermatogenesis [13]. Meanwhile, the modulation of microbiota was shown to control the autophagy machinery and counteract the toxicity [7]. The intestinal microbiota regulates the autophagy process through multiple mechanisms, including regulating protein metabolism [14] and inhibiting histone deacetylases [15]. Interestingly, the gut microbiome produces short chain fatty acids (SCFAs) in circulation, which act as signal molecules [16] to regulate the autophagy in different organs [17]. Acetate and propionate are the highest SCFAs in the colon and account for more than half of the SCFAs detected in the feces [16]. As a matter of fact, acetate and propionate were recognized as ligands for G protein coupled receptors (GPCRs), through which they induce peroxisome proliferator-activated receptor-γ coactivator-1 α (PGC-1α) [18]. PGC1α has recently been described as a master control of mitophagy as well as mitochondrial function and biogenesis [19].

Untapped pharmaceutical action of different natural and pharmacological substances are worth investigation [20]. RFX is a poorly absorbed antibiotic, which gives it the virtue of good bioavailability in the gut, so it is effective for the treatment of travelers’ diarrhea, digestive disorders, and extended to hepatic encephalopathy [21]. In addition, RFX has the ability to reduce pathogenic bacteria and eliminate local intestinal inflammation [21]. RFX was shown as a prominent factor in shaping the gut microbiome community structure [22], suggesting that RFX has autophagy modulation action. However, the potential of RFX as an off-target health benefit on malathion-induced disruptive autophagy and the corresponding testopathy needs investigation. 

In the present study, we hypothesized that RFX, through alleviation of malathion-induced dysbiosis and thus the increase in SCFAs level, is able to ameliorate malathion-induced testicular dysfunction. We hypothesize that the protective effect will be mediated via the induction of the antioxidant power and, thereby, autophagy and mitophagy. 

In the present study, we explored the potential of RFX to alleviate the dysregulation of the interaction among gut microbiota and environmental factors on one hand and aberrant cellular processes, such as antioxidant defense system, autophagy and mitochondrial functions, on the other, thereby preventing the development of testicular dysfunction.

## 2. Materials and Methods

### 2.1. Drugs and Chemicals

Malathion, a diethyl 2-dimethoxyphosphinothioylsulfanylbutanedioate of 97% Tc and 57% Ec, was purchased from El-Nasr Chemical Co.^®^ (Cairo, Egypt). RFX was provided as “Trencedia^®^”. Acetic acid, propionic acid, and butyric acid were purchased from Sigma-Aldrich (Copenhagen, Denmark). Primary and secondary antibodies were obtained from Abcam (Cambridge, MA, USA). Other chemicals and solvents were of the highest grade commercially available.

### 2.2. Animals and Experimental Design

This research protocol was approved by the Research Ethics Committee of the Faculty of Medicine, Ain Shams University (FMASU-REC), approval code: R51/2019. All animal procedures and care were carried out according to the general guidelines of the FMASU-REC, and they conformed to the guiding principles of the ARRIVE guidelines and were in accordance with the U.K. Animals (Scientific Procedures) Act, 1986, and associated guidelines, and the EU Directive 2010/63/EU for animal experiments.

Forty-eight adult male Sprague–Dawley rats (7 weeks of age) were obtained from the Nile Co. for Pharmaceuticals and Chemical Industries, Cairo, Egypt. The study was conducted in the Laboratory of Animals Research Center in the Faculty of Medicine, Ain Shams University. The rats were maintained under controlled temperature and 12 h light/12 h dark conditions for one week before the start of the experiments. They were allowed ad libitum access to standard laboratory feed and tap water. At 8 weeks of age and after acclimation, forty-eight male rats were divided into the following four groups of twelve animals each: (1) control rats and received corn oil (0.5 mL/kg via gastric lavage) daily, as vehicle for malathion, and saline (10 mL/kg via gastric lavage) daily, as vehicle for RFX; (2) rats treated daily with RFX (100 mg/kg in saline 10 mL/kg via gastric lavage) and corn oil (0.5 mL/kg via gastric lavage); (3) rats treated daily with malathion (50 mg/kg in corn oil 0.5 mL/kg via gastric lavage) and saline (10 mL/kg via gastric lavage); (4) rats treated daily with malathion (50 mg/kg in corn oil 0.5 mL/kg via gastric lavage) concurrently with a daily dose of RFX (100 mg/kg in saline 10 mL/kg via gastric lavage). All groups were a fed normal diet.

In order to determine which dose of RFX was more appropriate in malathion toxicity; two doses of RFX (50 and 100 mg/kg) were used for hormonal assessment and, depending on the results attained from this experiment, the proper RFX dose was selected and used for further mechanistic studies. RFX at a dose of 100 mg/kg was more effective, which coincided with previous study conducted by Kitagawa [23]. As for malathion, the dose was established following a preliminary study using three different concentrations (25 mg/kg, 50 mg/kg, 100 mg/kg) less than the LD_50_ of malathion in rats. The selected dose of malathion (50 mg/kg) achieved moderate toxicity with no lethality. Consistently, Geng and others used a dose of 50 mg/kg [24,25].

In each group, 6 rats were anesthetized by sodium pentobarbital (100 mg/kg, intraperitoneal injection) then sacrificed after two weeks, the remaining 6 rats in each group that completed the 28-day experiment period were anesthetized by sodium pentobarbital (100 mg/kg, intraperitoneal injection) and then were sacrificed by cervical dislocation, which is in line with euthanasia guidelines. Throughout the experiment, all animals were observed at least once a day for clinical signs of toxicity related to malathion exposure.

### 2.3. Bioaccumulation of Malathion in Testicular Tissues

Malathion analysis in testicular tissues was carried out on a gas chromatograph (Agilent GC-7890) (Santa Clara, California, CA, USA) equipped with a split/splitless injector system and flame ionization detector at 270 °C. A capillary column (DB5, 25 m × 0.32 mm i.d. and 0.25 µm film thickness) from SGE (Victoria, Australia) was used for the separation. The column oven temperature was initially held at 150 °C for 2 min, raised to 200 °C at 5 °C min^−1^, and held for 5 min. The injector temperature was set at 250 °C. The carrier gas was Nitrogen at a flow rate of 2 mL min^−1^ [26].

### 2.4. Physiological Assessment

Body weights of rats were recorded weekly during the study. After the animals were euthanized at 2 and 4 weeks, the testes were collected and weighed.

### 2.5. Blood Collection and Tissue Processing 

After day 14 and day 28, blood samples were collected from 6 rats in each group, then animals were sacrificed by rapid decapitation. Blood was withdrawn from the retro-orbital plexus and allowed to clot. Serum was separated by centrifugation at 3000× *g* for 15 min and used to evaluate testosterone and SCFA levels. After decapitation, the two testicles were quickly dissected, weighted, and immersed in a cooled (2–8 °C) 0.9% NaCl solution. One testicle was used for histopathology and transmission electron microscopy studies (Samples in Formaldhyde and Glutaldhyde, respectively). The second testicle was immediately frozen on dry ice and stored at −80 °C for biochemical analysis and protein expression by Western blot. Specimens of intestinal contents were collected and stored at −80 °C until used.

### 2.6. Sperm Collection and Evaluation

At the time of laparotomy after two and four weeks, the left epididymidis of each rat was collected and the caudal epididymis was used to prepare sperm suspension for measuring sperm counts, motility, and morphology [27,28]. Briefly, immediately after the sacrifice, the left caudal epididymis of each animal was homogenized in 2 mL of warm (37 °C) phosphate buffered saline (PBS), of which 20 μL was used for the evaluation of sperm mobility after placing it on a glass slide and covering it with a cover slip. The motility of spermatozoa was measured in terms of the percentage of motile spermatozoa in total spermatozoa, which was determined using a hemocytometer under a light microscope. The sperm count was expressed as million/mL. The sperm smear slides were made and stained with 2% eosin for examination of the morphological changes in sperms, for which a total of 200 sperm were scored under the microscope in 100× magnifications. Abnormalities were scored and classified as: heads deformed, doubled, detached, or pin shaped; and tails curved, looped, curled, or broken.

### 2.7. Testosterone Assay

Blood was collected from the retro orbital plexus of the rats. After standing for 1 h, the blood samples were centrifuged for 10 min at 3000 rpm (1600× *g*), and the serum samples were stored at −80 °C until analysis. The serum testosterone level was examined by ELISA kit (Cusabio Biotech, Wuhan, China), as serum samples were added to the appropriate wells with an antibody specific for testosterone and horseradish peroxidase (HRP) conjugated testosterone. After the addition of substrates, the absorbance was read at 450 nm [29].

### 2.8. SCFA by GC-MS

SCFAs (acetate, propionate, and n-butyrate) were extracted from samples using the Folch method (chloroform:methanol, 2:1, *v*/*v*) [30]. The GC–MS analysis was carried out using gas chromatography–mass spectrometry instrument stands with the following specifications: a TRACE GC Ultra Gas Chromatographs (THERMO Scientific Corp., Waltham, MA, USA), coupled with a thermo–mass spectrometer detector (ISQ Single Quadrupole Mass Spectrometer). The GC–MS system was equipped with a TR-5 MS column (30 m × 0.32 mm i.d., 0.25 μm film thickness). Analyses were carried out using helium as carrier gas at a flow rate of 1.0 mL/min and a split ratio of 1:10 using the following temperature program: 60 ^◦^C for 1 min; rising at 4.0 ^◦^C/min to 240 ^◦^C and held for 1 min. The injector and detector were held at 210 °C. Diluted samples (1:10 hexane, *v*/*v*) of 1 μL of the mixtures were always injected. Mass spectra were obtained by electron ionization (EI) at 70 eV, using a spectral range of *m*/*z* 40–450. The identification of the chemical constituents of the essential oil was deconvoluted using AMDIS software (www.amdis.net, accessed on 21 July 2020) and identified by its retention indices, mass spectrum matching to authentic standards (when available), and Wiley spectral library collection. The concentration of SCFAs in serum was calculated according to the peak area of acid.

### 2.9. Testes Malondialdehyde Measurement

Testes tissue samples were homogenized using a homogenization buffer containing 1.15% KCl solution and then centrifuged at 1500× *g* for 10 min. Then, the homogenate samples were added to a reaction mixture containing sodium dodecyl sulfate, acetic acid (pH 3.5), thiobarbituric acid, and distilled water [31]. After boiling the mixture for 1 h at 95 °C and centrifuging it at 3000× *g* for 10 min, the absorbency of the supernatant was noted using spectrophotometry at a 550-nm wavelength. The MDA concentrations are expressed in nmoles per mg tissue.

### 2.10. Catalase Assay

Testes tissues were homogenized in 50 mM phosphate buffer (pH = 7.4). The homogenate was centrifuged at 10,000 rpm for 10 min at 4 °C. Catalase (CAT) activity was estimated using the method of Aebi [32]. An amount of 100 μL tissue supernatant was added to a cuvette containing 1.5 mL of CAT mixture (H_2_O_2_ + 50 mM phosphate buffer). The reaction was started by the decomposition of H_2_O_2_ and CAT activity was measured using spectrophotometer at 240 nm. 

### 2.11. Assessment of Superoxide Dismutase (SOD) Activity

Testicular SOD activity was assessed biochemically [33]. In a spectrophotometric cuvette, 2.04 mL of 50 mM Tris buffer (pH-8.2), 20 µL of sample, and 20 µL of pyrogallol were taken and the absorbance was recorded in a spectrophotometer at 420 nm.

### 2.12. Histopathological Examination

Tissue samples from testes were fixed in 10% buffered formalin overnight, and then were washed under tap water and dehydrated through a graded series of ethanol (30–100%). The testicular tissues were later embedded in paraffin and were cut at a 5 μm thickness using a rotatory microtome. After dewaxing in xylene, the sections were passed through decreasing grades of alcohol and stained with hematoxylin. After that, the sections were gradually dehydrated up to the 70% alcohol and stained with eosin, after further dehydration up to absolute alcohol the sections were cleared with a clearing agent (xylene) and finally mounted with Dibutylphthalate Polystyrene Xylene DPX. The slides were observed under a light microscope for the histological changes and morphometrical examination [34].

To evaluate spermatogenesis or spermatogenic arrest, the mean testicular biopsy score (MTBS) was utilized [35]. This classification is a reliable technique to assess the proportion of normal and pathological tubules. Accordingly, a score of 1–10 was given to each tubule (Table 1). 

For histomorphometric analysis, the mean seminiferous tubule diameter (MSTD) and seminiferous epithelium thickness were evaluated according to a method described previously [36]. As for MSTD, two diameters at right angles to each other, passing through the center of the tubule were measured in µm. The height of the epithelium was measured in µm from seminiferous tubules that were round or nearly round.

### 2.13. Transmission Electron Microscopy (TEM)

A part of the testes was cut into small pieces (1 mm^3^), treated with 2.5% glutaraldehyde 0.1 N PBS at room temperature for one hour, and then fixed with osmium tetraoxide for another hour. Testes tissue was dehydrated in 70, 90 and 100% acetone (twice) for five minutes each, then dehydrated in 1:1 (acetone:resin) for five minutes and then embedded in epoxy resin. Observation for ultrathin slices (90 nm) was carried out using a transmission electron microscope, Jeol Jem- 1400, Peabodody, MA, USA.

### 2.14. Next Generation 16S rRNA Sequencing Method

Contents of the large intestine were frozen at −80 °C, mammalian tissue was lysed by mechanical disruption using a TissueLyser II (Qiagen, Venlo, The Netherlands). The microbial DNA was extracted using the PCR amplification kit (Ion Torrent. Thermo Fisher Scientific, Waltham, MA, USA). It includes 2 sets of primers that can be used to amplify the corresponding hypervariable regions of the 16S rDNA gene in bacteria: Primer set V2–4–8 and Primer set V3–6, 7–9. The regions of 16S rRNA genes were amplified and sequenced using the Ion PGM Sequencing 400 Kit on the Ion PGM platform system sequencer, following manufacturer’s instructions. The sequence data were preprocessed and analyzed using the “Ion 16S metagenomics analysis module” in Ion Reporter software (Thermo Fisher Scientific, Waltham, MA, USA). In brief, the R1 and R2 read pairs were joined and chimera sequences were removed. The operational taxonomic unit (OTU) picking was performed by using curated Greengenes database and premium curated Micro SEQ ID 16S rRNA reference database. The representative sequences of each OTUs were picked and taxonomy assignment was performed by using the Ion Universal Library Quantitation Kit. KRONA provided the graphical representation of the taxonomic classifications and the hierarchical structures. 

### 2.15. Western Blot Analysis for the Expression of LC3-II/I, P62, PINK1, PGC-α, TFAM, and Cleaved Caspase-3

Protein was extracted from the tissues using a mirVana PARIS kit, where cell disruption buffer was used to disrupt samples, and the resulting lysate was used for protein analysis. The lysate used in protein analysis was placed on ice for 5–10 min and centrifuged at 4 °C for 1–2 min. Protein concentrations were estimated by the Bradford method. Equal amounts of protein per lane were separated on 10% gels via SDS polyacrylamide gel electrophoresis and were electrophoretically transferred to polyvinylidenene difluoride (PVDF) membranes. Membranes were then incubated at room temperature for 2 h with blocking solution comprised of 5% nonfat dried milk in 10 mM Tris-Cl, Ph 7.5, 100 Mm NaCl, and 0.1% Tween 20. Membranes were incubated overnight at 4 °C with the indicated primary antibodies against β-Actin, Microtubule-associated protein 1A/1B-light chain 3 (LC3)-II/I (1:1000, Millipore), P62, PGC-1α (1:1000, Cell Signaling Technology, Danvers, MA, USA), Transcription Factor A, Mitochondrial (TFAM) (1:1000; Abcam), PINK1 (1:1000; Abcam), and cleaved Caspase 3 (1:1000, Cell Signaling Technology). Then, the membranes were incubated with a mouse anti-rabbit secondary monoclonal antibody conjugated to horseradish peroxidase at room temperature for 2 h. After each incubation, the membranes were washed four times with 10 mM Tris-Cl, Ph 7.5, 100 Mm NaCl, and 0.1% Tween 20 at room temperature. Chemiluminescence detection was performed with an Amersham detection kit according to the manufacturer’s protocol. The amount of the protein of interest was quantified by densitometric analysis using BioRad software, USA. Results were expressed as arbitrary units after normalization to β-Actin protein expression.

### 2.16. Statistics

Data were analyzed using one-way ANOVA (Prism 8, GraphPad, San Diego, CA, USA) followed by Bonferroni-corrected post hoc tests when appropriate. Pearson’s correlation was utilized to examine the potential associations between continuous variables. All results are presented as the means ± SEM. A threshold of *p* < 0.05 was considered statistically significant.

## 3. Results

### 3.1. Bioaccumulation of Malathion in Testicular Tissues

**Figure 1:**
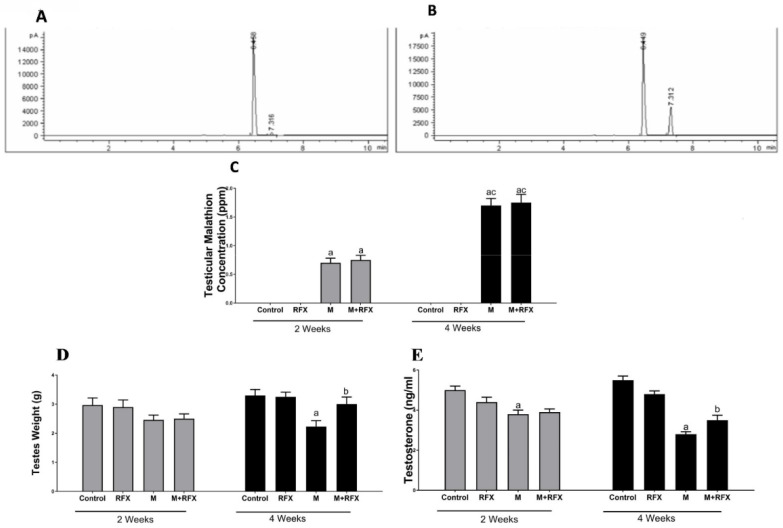
Testicular malathion concentration and the effects of RFX administration on testes weights and testosterone serum levels in rats. (**A**): Representative chromatogram of malathion concentration in testicular tissues after 2 weeks. (**B**): Representative chromatogram of malathion concentration in testicular tissues after 4 weeks. (**C**): Representative bars of testicular malathion concentration (ppm) after 2 and 4 weeks in different groups. (**D**): Testes weight (g) after 2 and 4 weeks in different groups. (**E**): Testosterone levels (ng/mL) after 2 and 4 weeks. Data are presented as the means ± SEM. a, significant (*p* < 0.05) versus control; b, significant (*p* < 0.05) versus the malathion group; c, significant (*p* < 0.05) versus the two weeks group; data were analyzed with one-way ANOVA followed by Bonferroni-corrected post hoc tests. RFX, rifaximin; M, malathion; M + RFX, malathion and rifaximin.

The testicular tissues of all studied groups after 2 and 4 weeks were analyzed for the concentration of malathion. The results revealed that longer exposure to malathion induced significantly higher concentration of malathion in the testicular tissues. The administration of RFX showed no significant change on testicular malathion levels when compared to the malathion group (Figure 1A–C).

### 3.2. Effects of RFX Administration on Body and Testes Weights in Rats Exposed to Malathion

Rats treated with malathion at a dose of 50 mg/kg for two and four weeks showed no clinical signs of toxicity or deaths. There was no significant difference in body weight between the different groups at the beginning, on day 14, and on day 28 (Table 2). As for the testes weight, there was no significant difference between the malathion group and the control group on day 14. However, on day 28, the absolute testicular weight of the malathion group decreased significantly compared to the control group (*p* < 0.05) (Figure 1D). Compared with the malathion group, RFX administration for four weeks significantly increased the absolute testicular weight (*p* < 0.05).

### 3.3. Effects of RFX Administration on Testosterone Serum Levels in Rats Exposed to Malathion

Compared with the control group, malathion exposure for two and four weeks significantly reduced the testosterone levels (Figure 1E). After treatment with RFX for four weeks, testosterone levels were significantly increased compared to the malathion group (*p* < 0.05). 

### 3.4. Effects of RFX Administration on Sperm Parameters in Rats Exposed to Malathion

**Figure 2:**
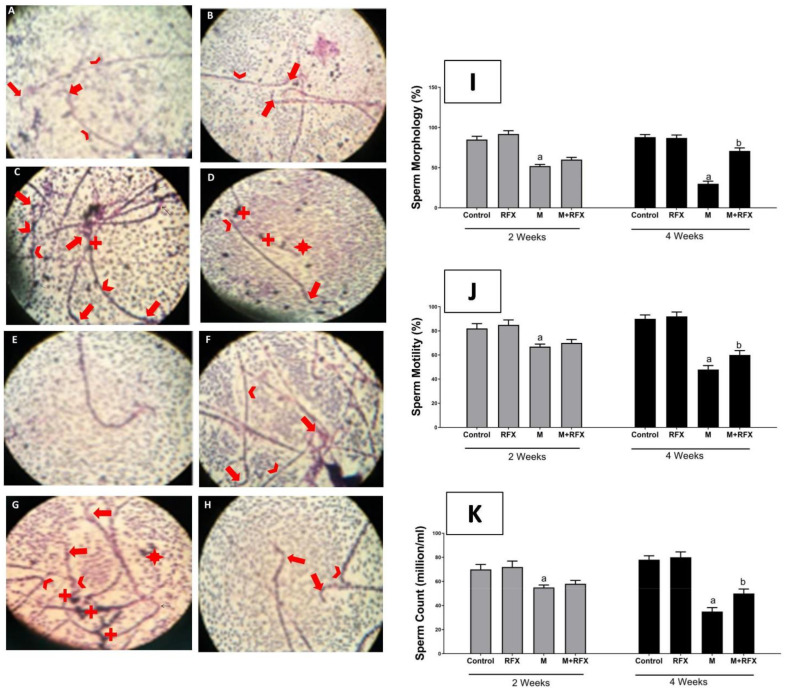
Effects of RFX administration on sperm parameters in rats exposed to malathion. (**I**): Representative bars of sperm morphology (%). (**J**): Representative bars of sperm motility (%). (**K**): Representative bars of sperm count (million/mL). Values are expressed as the means ± SEM. a, significant (*p* < 0.05) versus control; b, significant (*p* < 0.05) versus malathion group. One-way ANOVA followed by Bonferroni-corrected post hoc tests were performed. Sperm morphology was illustrated by microphotographs of the control group after 2 weeks (**A**), RFX group after 2 weeks (**B**), control group after 4 weeks (**E**), and RFX group after 4 weeks (**F**), all of which had normal heads (arrow) and tails (arrowhead). In M/2 w group (**C**), sperm were headless or had deformed heads, while some showed pinheads (arrow), and tails were curved or broken (arrowhead), in addition to cytoplasmic droplets (+). M + RFX/2 w group (**D**) shows headless sperm and detached heads (*), broken tails (arrowhead), and cytoplasmic droplets (+). Additionally, an intact head is shown (arrow). In the M/4 w group (**G**), sperm were non-vital with tails that are looped or curled (arrowhead), double headed or with deformed heads (arrow) and detached heads (*), in addition to massive cytoplasmic droplets (+). In the M + RFX/4 w group (**H**), sperm had intact heads (arrow) and there were no cytoplasmic droplets. Malformed heads with reduced hook were scarcely abundant (arrow).

Rats exposed to malathion for two and four weeks exhibited abnormal morphology (Figure 2I) and had lower motility (Figure 2J) and sperm count (Figure 2K) compared to the control group. The quantification of all abnormalities showed that the anomaly of sperm morphology was the most prominent. On the 14th day of the experiment, there was no significant difference between the RFX-treated rats and the malathion group.

After four weeks, the sperm motility and count of M + RFX-treated rats were significantly higher than that in the malathion group. The percentage of sperm dysmorphology in M + RFX-treated rats was statistically lower than that in the malathion group (*p* < 0.05) (Figure 2I). Sperm morphology was illustrated by microphotographs in all the control groups, demonstrating normal heads and tails (Figure 2A,B,E,F). The malathion groups showed a massive abundance of cytoplasmic droplets in addition to non-vital sperm with the head deformed or detached and the tail broken, looped, or curled (Figure 2C,G). On the other hand, M + RFX-treated rats for 4 weeks showed no cytoplasmic droplets and had some vital sperms (Figure 2H).

### 3.5. Histopathological Examination of the Testes

**Figure 3:**
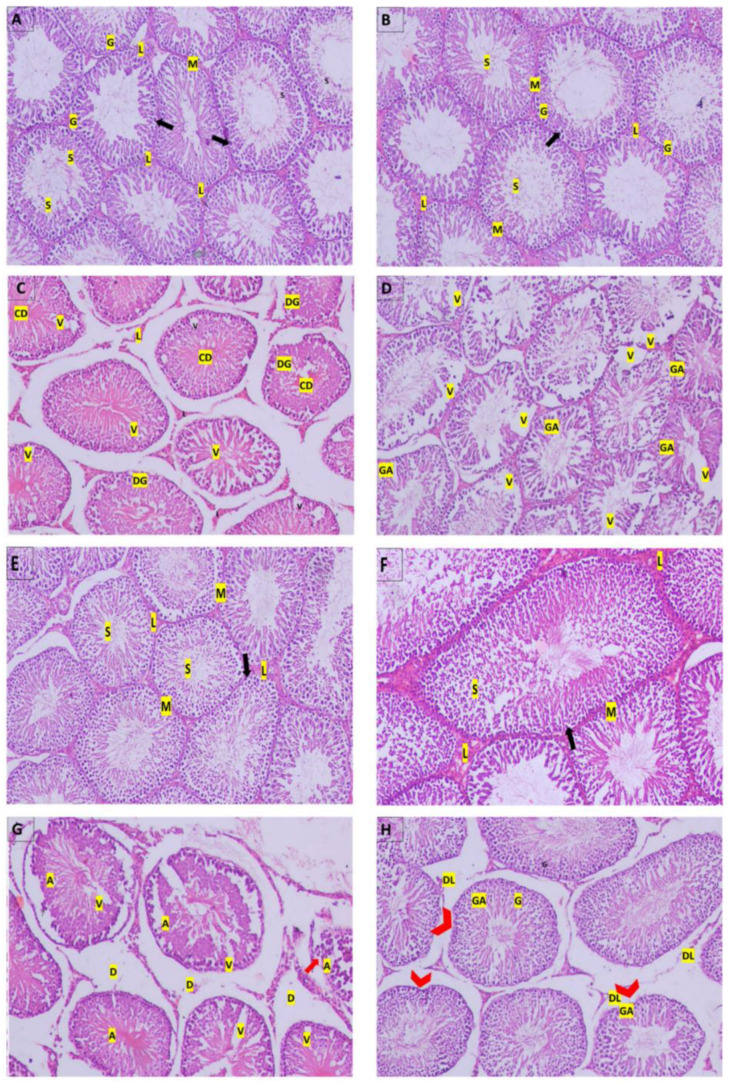
Photomicrographs of histological sections of rats’ testicular tissue by hematoxylin- and eosin. (**A**): Control group after 2 weeks; (**B**): RFX group after 2 weeks; (**E**): Control group after 4 weeks; (**F**): RFX group after 4 weeks; all show normal histological structure of rats’ testes with closely packed seminiferous tubules with Sertoli cells (arrow), spermatogenic cells (G), sperm (S), and were surrounded by clusters of peritubular myoid cells (M) and Leydig cells (L). (**C**): M/2 w group shows loosely packed necrotic seminiferous tubules with degenerated spermatogenic cells (DG), sperm (S), and cellular debris in the lumen, surrounded by expanded interstitial space that contained peritubular-degenerated Leydig cells (L). (**D**): M + RFX/2 w group had densely packed seminiferous tubules showing spermatogenic arrest (GA), predominantly at the level of spermatogonium, while others are vacant displayed disorganized spermatogenic cells (DG), few sperm (S), and were surrounded by peritubular myoid cells (M). (**G**): M/4 w group shows atrophied seminiferous tubules (A) with vaculation (V), the disruption of the interstitial connective tissue (D), and the detachments of the spermatogonia from the basement membrane (arrows). (**H**): M + RFX/4 w group shows partial recovery, as some of the seminiferous tubules are intact, revealing normal spermatogenic cells (G) with no vaculation and sperm (S) being abundant in the lumen; however, a few had irregular membranes (arrowhead) and showed spermatogenic arrest (GA). Leydig cells were mostly intact (L), but few were degenerated (DL). In addition, damage in the interstitial connective tissue was relatively contained.

Histopathological observation of the testicular tissues is shown in Figure 3. The control group, after 2 and 4 weeks (Figure 3A,E), and the RFX group, after 2 and 4 weeks (Figure 3B,F), both showed normal testicular structure where closely packed seminiferous tubules had Sertoli cells, intact spermatogenic cells, plenty of sperm, and were supported with cluster of peritubular myoid cells and Leydig cells. On the other hand, after 2 weeks of malathion exposure (Figure 3C), there was a mild disruption of the spermatogenesis process, which extended to severe degenerative changes after 4 weeks (Figure 3G) to involve the atrophy of the seminiferous tubules and the degeneration of spermatogenic and Leydig cells. Cotreatment with RFX resulted in the amelioration of the toxic effects of malathion, as revealed by the abundance of normal spermatogenic cells and sperm (Figure 3D,H). Still, few seminiferous tubules had irregular membranes and showed spermatogenic arrest.

### 3.6. Histomorphometric Analysis of the Seminiferous Tubules

Malathion treatment for 2 and 4 weeks resulted in decreased spermatogenic cell layers and lowered the density of spermatozoa in the lumen and seminiferous tubules lumen. Accordingly, the mean testicular biopsy score was significantly lower after 2 and 4 weeks of treatment with malathion compared to the control group (*p* < 0.05). A significant increase in the MTBS value after RFX administration was observed compared to the malathion group (*p* < 0.05). 

**Figure 4:**
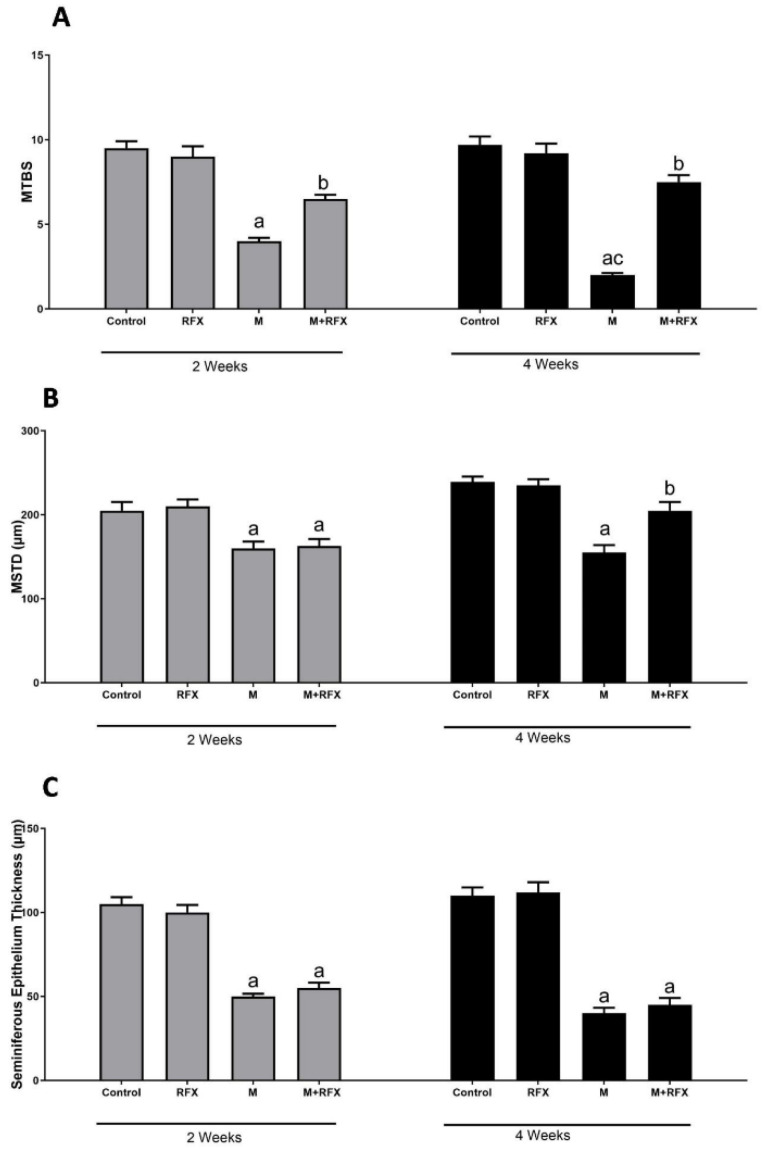
Histomorphometric analysis of the seminiferous tubules. (**A**): Changes in the mean testicular biopsy scores (MTBS) of different groups. (**B**): Changes in the seminiferous tubule diameter (MSTD in µm) of different experimental groups. (**C**): Changes in the seminiferous epithelium thickness in µm of different experimental groups. Data are presented as the means ± SEM. a, significant (*p* < 0.05) versus control; b, significant (*p* < 0.05) versus the malathion group; c, significant (*p* < 0.05) versus the two weeks group; data were analyzed with one-way ANOVA followed by Bonferroni-corrected post hoc tests. RFX, rifaximin; M, malathion; M + RFX, malathion and rifaximin.

As for the mean seminiferous tubule diameter (MSTD), it was significantly reduced when compared to the control rats, while it was significantly increased when RFX was coadministered for 4 weeks (Figure 4). On the other hand, no significant change was found with respect to the thickness of the epithelium between the malathion groups and the M + RFX groups (*p* > 0.05).

### 3.7. Electron Microscopical Examination of Testicular Tissue

**Figure 5:**
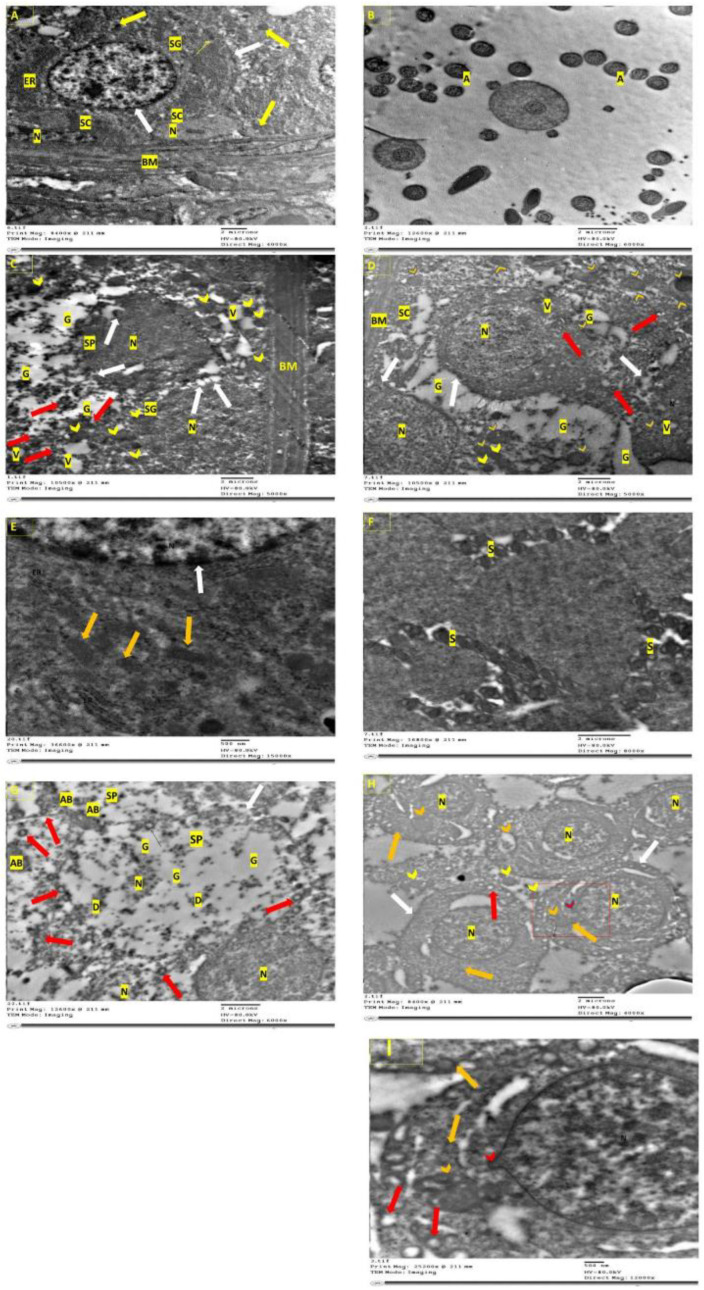
Ultra-structure of testes by Transmission Electron Microscopy. (**A**): Control group with basement membrane, which has myoid cells, and resting in which are spermatogonium with a dome-shaped nucleus, nucleolus, ER, golgi, and large number of mitochondria with intact cristae (yellow arrow). Sertoli cell (SC) exhibited elongated nucleus, nucleolus. Intact nuclear and cytoplasmic membrane (white arrow). (**B**): Negative control after 2 weeks showing spermatozoa with normal head and containing a nucleus and axoneme (A) with a typical “9  +  2” microtubule structure (nine pairs of peripheral and two central microtubules). (**C**): M/2 w group with irregular basal (BM) and apoptotic spermatogonia (SG) with the condensation and margination of the dome-shaped nucleus (N); the blipping of nuclear and cytoplasmic membrane (white arrow), degenerated endoplasmic reticulum, damaged mitochondria (red arrow), or swollen mitochondria with loss of cristae (yellow arrowhead), extensive vaculation (V), and apoptotic spermatocyte (SP) that also shows the blipping of nuclear and cytoplasmic membranes (white arrow) and wide cytoplasmic gaps (G). (**D**): M + RFX/2 w group with irregular basal membrane (BM) and damaged Sertoli cells (SC) resting on it. Additionally, primary spermatocytes (PS) with irregular, damaged nuclear and cytoplasmic membrane (white arrow), and damaged mitochondria (red arrow), all of which indicate apoptosis. Signs of autophagy involved cytoplasmic vacuoles (V) and multiple phagosomes with mitochondria inside (orange arrowhead). (**E**): Control group after 4 weeks showing a spermatocyte nucleus with an intact nuclear membrane (white arrow), intact mitochondria with cristae (orange arrow), and a well-organized endoplasmic reticulum (ER). (**F**): Negative control after 4 weeks showing the successful production of spermatozoa (S). (**G**): M/4 w group shows degenerate spermatocyte (SP) with a damaged cell membrane (white arrow), pyknotic nucleus (N), empty mitochondria (red arrow), wide cytoplasmic gaps (G) degenerated cellular components (D), and apoptotic bodies (AB). (**H**): M + RFX/4 w group shows partial recovery with primary spermatocytes, which have definite cytoplasmic (NM) and nuclear membrane (NM), gaps around the nucleus have some blebbing (red arrowhead) in the NM as a step towards apoptosis. Many intact mitochondria (orange arrow), with some empty (red arrow), while a few are swollen (yellow arrowhead). Some autophagosomes can be spotted (orange arrowhead). (**I**): Higher magnification of M + RFX/4 w.

The ultra-structure of the rats’ testes was shown in Figure 5. The control group after 2 and 4 weeks (Figure 5A,E), and negative control after 2 and 4 weeks (Figure 5B,F), all showed normal ultra-testicular structure in form of intact myoid, spermatogonia, and Sertoli cells. Structural abnormalities after malathion exposure for 2 and 4 weeks (Figure 5C,G) involved extensive apoptosis, which has the hallmark of degenerated mitochondria, while autophagy was highly limited. On the other hand, after 2 and 4 weeks of cotreatment with RFX (Figure 5D,H), autophagy was predominant, mitochondria were restored, and apoptosis was diminished.

### 3.8. Effects of RFX Administration for Four Weeks on the Abundance of Intestinal Flora in Rats Exposed to Malathion

We performed next generation sequencing analysis of 16S rRNA at the end of the experiment to determine the abundance of intestinal flora associated with malathion- and RFX-treated rats. 

**Figure 6:**
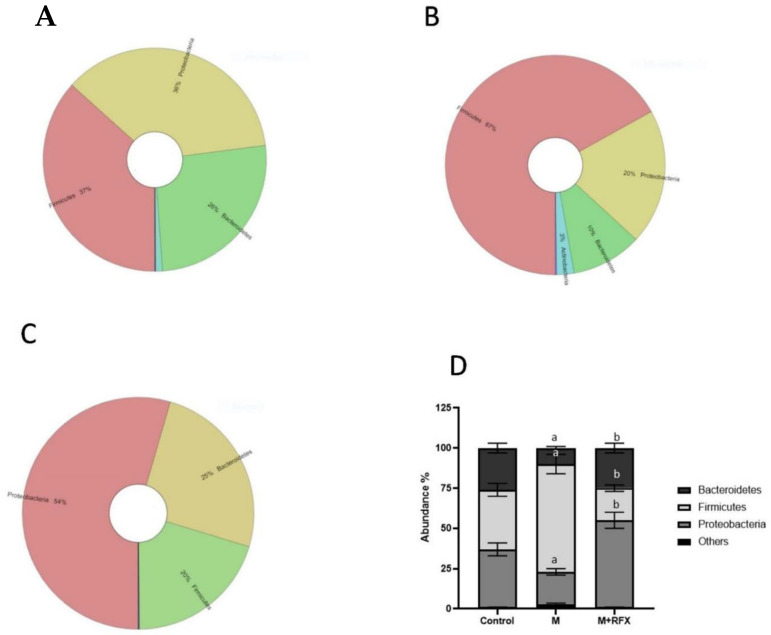
Effects of RFX treatment on the microbial diversity of the gut microbiota at the phylum level in rats exposed to malathion. Krona charts showing the phylum levels in the control group (**A**), malathion group (**B**), and malathion+rifaximin group (**C**). (**D**): Representative stacked column for the relative abundance of gut microbiota at the phylum level (Proteobacteria, Firmicutes, Bacteroidetes) in the three rat groups. Data are presented as the means ± SEM. a, significant (*p* < 0.05) versus control; b, significant (*p* < 0.05) versus the malathion group; data were analyzed with one-way ANOVA followed by Bonferroni-corrected post hoc tests. RFX, rifaximin; M, malathion; M + RFX, malathion and rifaximin.

Figure 6 showed the krona classification chart at the phylum level and the corresponding stacked bar chart (Figure 6D) for the relative abundance of each phylum in the three groups. The main phyla in the gut microbiota were Bacteroidetes, Firmicutes, and Proteobacteria. Comparing the malathion group to the control rats, there was a significant increase in relative abundance of Firmicutes (67 vs. 20%, *p* < 0.05) and reduction in Bacteroidetes, and Proteobacteria (*p* < 0.05). RFX treatment for 4 weeks significantly attenuated a malathion-induced increase in Firmicutes and decrease in Bacteroidetes and Proteobacteria. 

The ratio of Firmicutes to Bacteroidetes (Firm/Bac ratio) was significantly increased upon malathion exposure (*p* < 0.05), which was mitigated by RFX treatment. 

**Figure 7:**
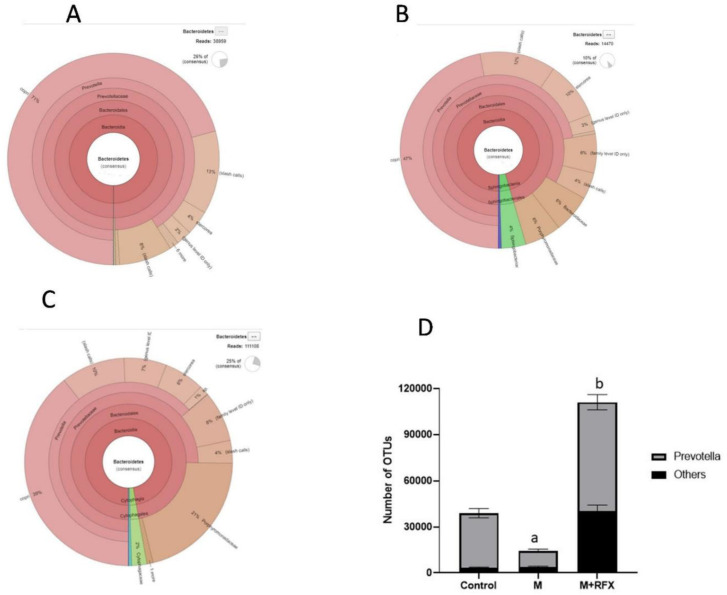
Changes in bacterial genus Prevotella of the phylum Bacteroidetes after RFX intervention in rats exposed to malathion for four weeks. Krona charts showing the genus Prevotella in the control group (**A**), malathion group (**B**), and malathion+rifaximin group (**C**). (**D**): Representative stacked column for the number of OTUs of the significantly changed bacterial genus Prevotella of the phylum Bacteroidetes in the three rat groups. Data are presented as the means ± SEM. a, significant (*p* < 0.05) versus control; b, significant (*p* < 0.05) versus the malathion group; data were analyzed with one-way ANOVA followed by Bonferroni.

Furthermore, krona classification charts and corresponding stacked bar charts showed the major composition and the relative abundance of gut microbiota at the class, order, and genus levels in rats’ intestinal content. After malathion exposure the main SCFA producing phylum, Bacteroidetes was down-represented (Figure 6B) as well as the key SCFA producer genus, Prevotella (Figure 7). 

**Figure 8:**
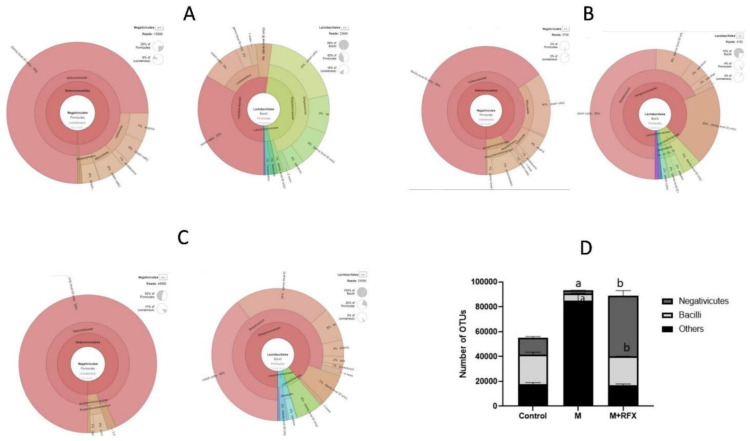
Changes in bacterial classes (Negativicutes and Bacilli) of the phylum Firmicutes after RFX intervention in rats exposed to malathion for four weeks. Krona charts showing Negativicutes and Bacilli levels in the control group (**A**), malathion group (**B**), and malathion+rifaximin group (**C**). (**D**): Representative stacked column for the number of OTUs of significantly changed bacterial classes (Negativicutes and Bacilli) of the phylum Firmicutes in the three rat groups. Data are presented as the means ± SEM. a, significant (*p* < 0.05) versus control; b, significant (*p* < 0.05) versus the malathion group; data were analyzed with one-way ANOVA followed by Bonferroni-corrected post hoc tests. RFX, rifaximin; M, malathion; M + RFX, malathion and rifaximin.

Notably, malathion administration down-represented the prominent classes that produce SCFA in the Firmicutes, namely, Negativicutes and Bacilli (Figure 8), involving their SCFA producers genera, Phascolarcobacterium, Streptococcus, and Lactobacillus. 

As shown in Figure 8, concomitant treatment of rats with RFX for 4 weeks together with malathion treatment induced significant increases in the abundance of Bacteroidetes, Negativicutes and Lactobacillales when compared to controls. Additionally, RFX treatment countered malathion-induced decreases in the abundance of Phascolarcobacterium, Streptococcus and Lactobacillus. 

### 3.9. Effects of RFX Administration on SCFAs in Serum in Rats Exposed to Malathion

**Figure 9:**
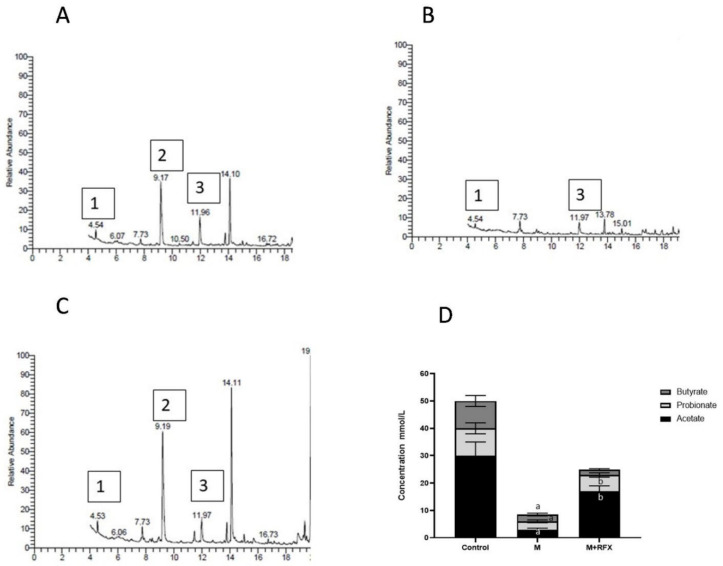
Modulation of short chain fatty acids in serum after RFX intervention in rats exposed to malathion for four weeks. Gas chromatogram showing acetic acid (1), propionic acid (2) and butyric acid (3) in the control group (**A**), malathion group (**B**) and malathion+rifaximin group (**C**). (**D**): Representative stacked column for the concentration of SCFAs in serum (mmol/L) as calculated according to the corresponding peak area of acid in gas chromatogram. Data are presented as the means ± SEM. a, significant (*p* < 0.05) versus control; b, significant (*p* < 0.05) versus the malathion group; data were analyzed with one-way ANOVA followed by Bonferroni-corrected post hoc tests. RFX, rifaximin; M, malathion; M + RFX, malathion and rifaximin.

As compared to the control group, the levels of the serum acetic and butyric acid were significantly lower in the malathion group, while the propionic acid was undetected (Figure 9). Compared with the malathion group, RFX administration for four weeks significantly increased the levels of the serum acetic and propionic acid (*p* < 0.05). As for the butyric acid levels, there was no significant differences between the malathion group and the M + RFX group (Figure 9).

### 3.10. Effects of RFX Administration on Serum and Testicular Oxidative Stress Markers in Rats Exposed to Malathion

**Figure 10:**
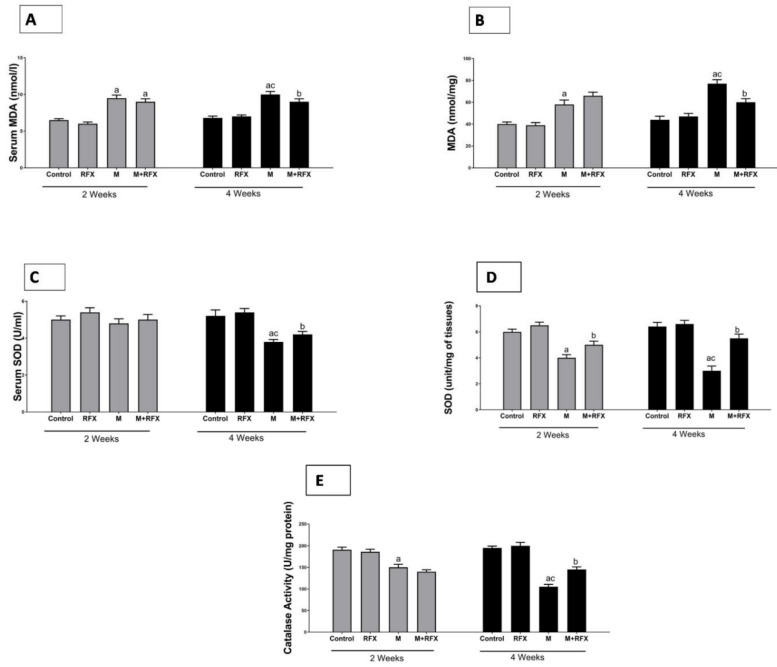
Effects of RFX administration on serum and testicular oxidative stress markers in rats exposed to malathion for four weeks. (**A**): Serum MDA (nmoles/L) after 2 and 4 weeks in different groups. (**B**): Testicular MDA (nmoles per mg tissue) after 2 and 4 weeks in different groups. (**C**): Serum SOD (unit/mL) after 2 and 4 weeks in different groups. (**D**): Testicular SOD (unit/mg of tissues) after 2 and 4 weeks in different groups. (**E**): Testicular catalase (unit/mg protein) after 2 and 4 weeks in different groups. Data are presented as the means ± SEM. a, significant (*p* < 0.05) versus control; b, significant (*p* < 0.05) versus the malathion group; c, significant (*p* < 0.05) versus the two weeks group; data were analyzed with one-way ANOVA, followed by Bonferroni-corrected post hoc tests. RFX, rifaximin; M, malathion; M + RFX, malathion and rifaximin.

The activities of serum and testicular SOD and testicular CAT in the malathion-treated rats at two and four weeks were significantly lower than that in the control group (*p* < 0.05), while MDA levels were significantly higher. The administration of RFX for two and four weeks significantly increased the activities of SOD and CAT in comparison to the malathion group, whereas the levels of MDA were decreased after four weeks of RFX administration (Figure 10).

### 3.11. Impact of malathion and RFX on Autophagy, Mitophagy and Mitochondrial Biogenesis

**Figure 11:**
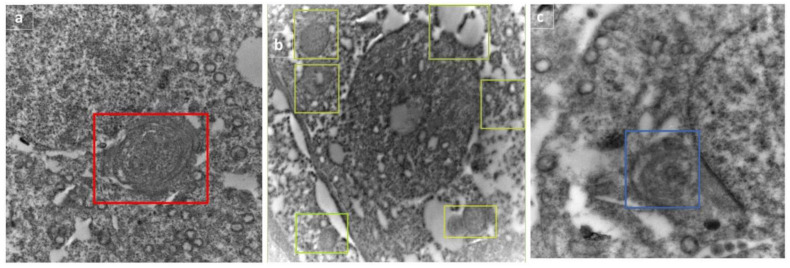
Selected micrographs of rats treated with RFX for two weeks after exposure to malathion show the assembly and progression of autophagic vesicles, indicating active autophagy flux in form of autophagosome (represented by box) (**a**), autophagosome–lysosome fusion (shown in boxes) (**b**), and autolysosome (shown in box) (**c**).

The autophagic dynamic was monitored by electron microscope in rats treated with RFX for two weeks after exposure to malathion. Selected micrographs showed the assembly and progression of autophagic vesicles in form of autophagosome, autophagosome-lysosome fusion, and autolysosome (Figure 11). 

To confirm active autophagic flux, the validation of LC3-II/I and P62 was performed by Western blot. LC3-II/I is an indicator of autophagy induction as it marks the number of autophagosomes. In the present study, LC3-II/I expression was significantly suppressed (−1.7- and −2-fold change) after malathion exposure for two and four weeks as compared to control groups (*p* < 0.05). When RFX was administered for two and four weeks, LC3-II/I expression was enhanced by 1.6- and 1.8-fold, respectively (*p* < 0.05) (Figure 13A). On the other hand, P62 showed autophagosome disintegration, thereafter, demonstrating an unusual buildup of P62 signifies impaired autophagic flux (Figure 13B).

**Figure 12:**
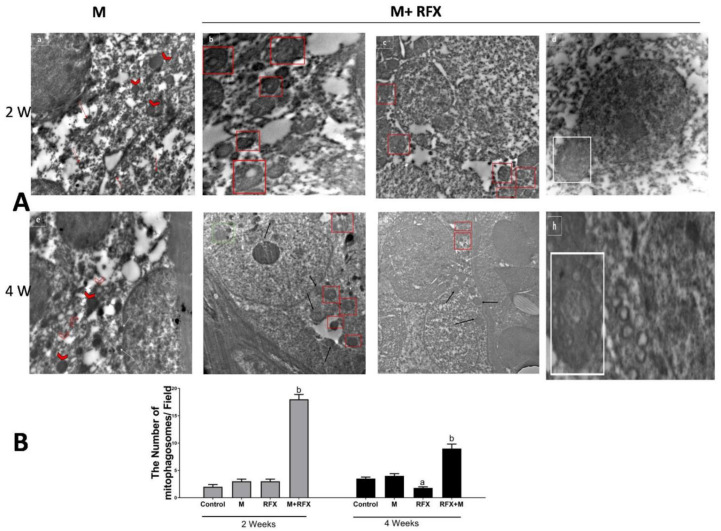
(**A**) Electron microscopic analysis of mitophagy in the malathion group and RFX treated groups. (**a**): Selected micrograph of rats exposed to malathion over two weeks clearly show the absence of autophagic vesicles, meanwhile some mitochondria exhibit a marked swelling (arrowhead); the rest of mitochondria are evidently impaired, with an absent double membrane; distorted cristae; and a noticeable reduction in electron-dense granules in the intramitochondrial matrix (red arrow). (**b**–**d**): Selected micrograph for rats treated with RFX for two weeks after exposure to malathion, where in (**b**,**c**), cells show various degree of damage with an abundance of mitophagosomes (red square), while in (**d**) typical autolysosome-enveloping mitochondria is visible (white square). (**e**): Selected micrograph for rats exposed to malathion for four weeks show diffused cellular damage, where mitochondria are visibly fewer in number and are mostly remnants of mitochondria (empty arrowhead) in addition to the swelling. Lysosomes (while arrow) are not involved in autolysosomes. (**f**–**h**): Selected micrograph for rats treated with RFX for four weeks after exposure to malathion, where, as seen in (**f**,**g**), cells exhibit various degree of retrieved vitality with an abundance of normal new intact mitochondria (black arrow), mitophagosomes (red square), lysosomes approximate to mitophagosomes (white arrow), and lysosomes in fusion with mitophagosome (green square), while in (**h**), typical autolysosome-enclosing mitochondria are noticeable (white square). (**B**) Quantification of mitophagosomes. The number of mitophagosomes/field was determined in electron micrographs. Data are expressed as means ± SEM.

Electron microscopy was employed to witness mitophagy. After malathion exposure for 2 and 4 weeks, autophagic vesicles were not detected, meanwhile diffused cellular and mitochondrial damage was evident. When rats were treated with RFX for 2 weeks and 4 weeks, mitophagosomes were abundant, autolysosome enveloping mitochondria were visible, and cells retrieved vitality (Figure 12A). For analysis of mitophagosomes, 10–20 fields were randomly selected from each animal from three different animals per group and were used for counting the mitophagosome [37]. The count of total mitophagosomes per field using electron micrographs showed a significant increase in the number of mitophagosomes after RFX treatment (*p* < 0.05) (Figure 12B).

**Figure 13:**
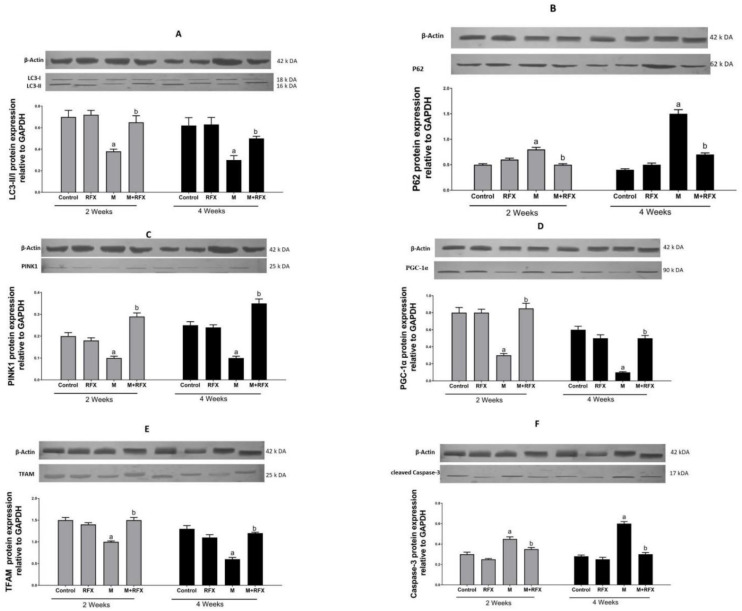
Effect of RFX administration on protein expression of LC3-II/LC3-I (**A**), P62 (**B**), PINK1 (**C**), PGC-1α (**D**), TFAM (**E**), and cleaved Caspase-3 (**F**) in the testicular tissue of rats exposed to malathion as shown by Western blot analysis. Bars represent protein expression levels relative to GAPDH. Values are expressed as the means ± SEM. a, significant (*p* < 0.05) versus control; b, significant (*p* < 0.05) versus malathion group; one-way ANOVA followed by Bonferroni-corrected post hoc tests were performed.

PTEN-induced putative kinase 1 (PINK1) is a serine/threonine mitochondrial kinase that plays a key role in directing the damaged mitochondria to the lysosomes, wherein the mitochondrial components are degraded and recycled (mitophagy). In this study, PINK1 protein level in the malathion group was significantly lower (*p* < 0.05) than that in the control group. The administration of RFX for two weeks significantly enhanced (*p* < 0.05) the PINK1 protein level (Figure 13C).

PGC-1α is the main transcription factor in charge of the formation of more mitochondria through increasing the expression of the gene encoding the key activator of the mitochondrial gene transcription, TFAM. In the present study, the protein expression of PGC-1α (Figure 13D), together with the expression of TFAM (Figure 13E), were inhibited (5 and 4 fold, respectively, *p* < 0.05) after malathion exposure. When RFX was administered, the expression levels of PGC-1α and TFAM were remarkably increased (4 and 3 fold, respectively, *p* < 0.05). 

### 3.12. Induction of Testicular Cells Apoptosis by Malathion and the Inhibitory Effect of RFX

Caspase-3 is a key executer of apoptosis, responsible for the proteolytic cleavage of critical proteins, leading to cell shrinkage, membrane blebbing, and DNA fragmentation. Therefore, Caspase-3 activation was examined in order to investigate the extent of apoptosis (Figure 13F). Compared with the control group, rats exposed to malathion for two and four weeks had higher a protein expression of cleaved Caspase-3 (1.5- and 2-fold, respectively, *p* < 0.05). Compared with the malathion group, when rats were administered RFX for two and four weeks, they had 1.3- and 2-fold less Caspase-3, respectively (*p* < 0.05). 

## 4. Discussion

The significant decline of male testicular function over time is produced by chronic exposure to environmental contamination, which elicits oxidative stress and contributes to cell damage. Over the years, it has been emphasized that intestinal flora is involved in the pathogenesis of multiple toxicological disorders and in treatment response [38]. 

In this study, malathion-induced testicular toxicity was evident in terms of reduced testicular weight, sperm count and motility, and serum testosterone level. Consistently, malathion was described as an endocrine disruptor that decreased reproductive performance [3,39]. In the current study, malathion administration for 4 weeks induced modification in the composition of microflora, as there was drop in the abundance of *Lactobacillus* spp., *Bacteroides* spp., *Prevotella* spp., *Streptococcus* spp., and *Phascolarctobacterium succinatutens*. It is of note that these bacteria are populations of SCFA-producing microflora. In normal conditions, SCFA-producing bacteria prevent excessive growth of pH-sensitive pathogenic bacteria, such as Enterobacteriaceae and Clostridia. The Bacteroidetes phylum mainly produces acetate and propionate, whereas the Firmicutes phylum has butyrate as its primary metabolic end product [40]. Our data showed that Enterobacteriaceae and Clostridia exhibited overgrowth. A previous report found that malathion altered the microbial community structure and tended to increase the risk for pathogen invasion [41]. We identified a significant decrease in SCFA production after malathion exposure. Apart from their intestinal role, SCFAs produced by the microbiota can be found in peripheral blood, where they are taken up by organs, as they act as signal molecules [16]. Therefore, it is plausible to consider that gut dysbiosis, resulting in the dysregulation of SCFA production, increases the susceptibility of testicular tissues to malathion toxicity. 

In order to obtain these insights, we studied the histopathological and biochemical changes in response to malathion toxicity at two time points: two and four weeks. After two weeks, histopathological analysis showed that malathion exposure induced seminiferous tubular necrosis, the degeneration of the germinal epithelium and the accumulation of the debris of germ cells in in the lumen. Furthermore, after four weeks of malathion administration, histopathological studies showed that the necrosis of seminiferous tubules was extended to complete atrophy. Interestingly, when we used TEM to examine the testicular tissues after two or four weeks, we observed that malathion exposure was accompanied mainly by active apoptosis, and we did not find any evidence of autophagy or mitophagy. Biochemical analysis of testicular tissues showed that malathion toxicity increased MDA levels and reduced the activity of SOD and CAT antioxidants. Earlier studies showed that malathion exposure triggered oxidative toxic stress, which contributed to decreased sperm motility, viability, and count [42]. Additionally, we reported extensive apoptosis, as supported by the elevated protein expression of Caspase-3. In line with the current findings, data from many reports clearly indicated the apoptotic effect of malathion [43]. Interestingly, we found that malathion impaired mitophagy as it inhibited the protein expression of PTEN-induced putative protein kinase 1 (PINK1). PINK1 ignited one of the most characterized mitophagy pathways to date. The inhibition of PINK1 designates the overwhelming of the autophagy mechanism due to excessive oxidative stress, leading to mitochondrial dysfunction [44,45]. In the absence of protection by autophagy, it could be suggested that the testicular apoptosis was the product of the surge of reactive oxygen species generated by malathion, known for their ability of apoptosis induction via mitochondria-dependent and -independent pathways [4]. Recently, it was discovered that malathion-induced toxicity via the impairment of the mitochondrial function induced apoptotic cell death [5]. 

The search for a safe, long-term remedy is of prime importance to overcome the limited effectiveness and adverse side effects of conventional drug therapy. RFX, being an unabsorbed antibiotic, has no systemic effects and good safety profile [46]. Our results show that RFX treatment significantly increased the weight of testes as well as the sperm activity and the serum testosterone level, indicating that RFX can resist testicular damage caused by malathion. Intriguingly, RFX induced a massive increase in the population of acetate-producing bacteria, such as *Lactobacillus* spp., *Bacteroides* spp., *Prevotella* spp., and *Streptococcus* spp, as compared to malathion-exposed rats. Moreover, the abundance of the propionate producing bacteria, *Phascolarctobacterium succinatutens* and *Negativicutes* spp., was enriched by RFX. 

RFX administration was able to increase the order of Bacteroidales, thereby reducing ethanol-induced liver damage [23]. In another report, RFX induced a significant increase in Lactobacilli in different gastrointestinal and liver diseases [47]. 

Following RFX administration, there was a significant increase in acetic and propionic acid levels in the serum. This effect, together with the testicular toxicity ameliorating effects, suggest that the mechanisms underlying RFX efficacy involve direct antibiotic action and the modulation of bacterial products. In the current study, RFX treatment was associated with an increased expression of peroxisome proliferator-activated receptor-gamma coactivator-1 alpha (PGC-1α), following malathion exposure for 2 and 4 weeks. To our knowledge, the association of RFX with PGC-1α has not been reported before. On the other hand, several lines of evidence indicated that SCFA enhanced the expression levels of PGC-1α as they can act as signaling molecules and bind to G-protein-coupled receptor 43 (GPR43), also known as a free fatty acid receptor 2 (FFAR2) [48,49]. Interestingly, FFAR2 exhibits the highest rate of expression in the testicular tissues [50]. Previously, PGC-1α has been implicated as a protective factor in experimental models of testicular toxicity [51,52]. PGC-1α is an upstream activator of mitophagy and mitochondriogenesis; the cellular survival processes [53,54].

Treatment with RFX for two weeks resulted in the induction of autophagy and inhibition of apoptosis as indicated by electron microscopic examination and biochemical analysis. These findings were verified by the Western blot analysis of LC3-II/I ratio, which was upregulated while Caspase-3 was found to be downregulated. LC3-II was initially present in the cytoplasm as microtubule-associated light chain 3 (LC3-I), but when autophagy was activated, it became phosphatidylethanolamine (PE)-conjugated membrane-bound form (LC3-II), which was finally recruited into the autophagosome; a prerequisite for the engulfment of the cargo [55]. Autophagy is an indispensable intracellular process that purges oxidative stress through clearing up damaged organelles, while using them as an energy source for recovered cells [56]. Therefore, the elimination of damaged mitochondria through mitochondrial autophagy (mitophagy) during chronic exposure to toxins could serve as a cellular adaptive mechanism to counteract testicular injury [57].

We sought to determine whether mitophagy was specifically induced after two weeks of RFX intake. In contrast to malathion, we found the increased protein expression of PINK1. Recent studies found that PINK1 can serve as a molecular sensor for the mitochondrial damage where it becomes stabilized on the outer mitochondrial membrane. The accumulation of PINK1 initiates mitophagy by recruiting protein responsible for ubiquitinating outer mitochondrial membrane proteins, allowing autophagy receptors to recognize labeled mitochondria [58]. Multiple studies have declared that PGC-1α is upstream to the activation of the bulk autophagy, as identified by LC3-II/I ratio, and to mitophagy, as recognized by PINK1 [59,60]. 

It is of note that the level of MDA in the testicular cells after two weeks of treatment with RFX was as high as in the malathion group, while the level of SOD was significantly lower, indicating the onset of improvement in oxidative stress and promising more significant results in the longer term. Therefore, we pursued the cyto-protective effects of RFX administration after four weeks of malathion administration. Because male gonads have a high energy requirement, mitochondrial health and function were our primary targets. TEM imaging after four weeks of treatment with RFX showed a large number of newly produced intact mitochondria, which were the hallmark of recovered seminiferous tubules. To further evaluate the protective effects of RFX after four weeks of malathion administration, a marker of new mitochondria formation was determined. In this regard, the protein expression of TFAM, which regulates mitochondrial genome replication and transcription [61,62], was found to be upregulated. Functional mitochondria, which are present in the tail of sperms are critical for sperm survival, motility maturation, and capacitation. The high demand of sperm cells for energy provided by mitochondria makes them particularly susceptible to toxins [63]. In the meantime, we found a significant decreased in the level of MDA and a significant increase in those of SOD and CAT, demonstrating successful cellular defense.

It worth noting that after exposure to malathion we found that the levels of butyrate-producers, such as Ruminococcaceae, Lachnospiraceae, and Faecalibacterium prausnitzii, decreased and RFX had no positive effects on them. Butyrate-producing bacteria play pivotal roles in inhibiting inflammation and modulating gut epithelium permeability [64]. In addition, RFX had no effect on butyrate serum levels. The limited effect of RFX on butyrate-producing bacteria may explain the incomplete recovery of testicular tissues.

In the current study, the state-of-the-art with RFX in the treatment of testicular disease proved the potential efficacy of RFX beyond conventional therapeutic targets. Over the past decade, extensive studies have expanded the application of RFX from gastrointestinal diseases, such as small intestine bacterial overgrowth, inflammatory bowel disease, and colonic diverticular disease [21], to encompass severe hepatic disorders. In this regard, RFX had valuable effects on cirrhosis [65] and ameliorated liver fibrosis [66] through the gut–liver axis in numerous experimental and clinical studies. Moreover, RFX was used off-label for the treatment of hepatic encephalopathy until it was approved in 2010 [67]. Recently, RFX has demonstrated an improvement in neurodegeneration in Alzheimer’s disease (AD) through the modulation of the gut microbiome [68]. In the current study, RFX was applied to male infertility, exploiting the non-traditional action of RFX on microbiota, and providing an insight into gut–testes axis. Lately, modifying gut microbiome provided the curation of male infertility in T1D patients [69]. Regarding the mechanism of the action of RFX, preclinical and clinical studies provided evidence for anti-inflammatory and immune modulator effects [70]. In the present study, we estimated the gut microbiome in the colon, examined SCFA metabolites in serum, and addressed the molecular mechanism of RFX in testicular tissues. Moreover, the novelty of our study lies in the dissection of groups of rats, where, utilizing malathion and RFX, activated mitophagy was found in a two-week group and the synthesis of new mitochondria was exhibited in a four-week group.

The limitations of this study involve using surrogate markers for apoptosis and autophagy, therefore future studies to explore the full panel of markers are recommended. It is also warranted to study the therapeutic effect of RFX in response to longer-term toxicity of various toxicants. The use of RFX in female infertility worth studying. Given the limited effect of RFX on butyrate-producing bacteria, we propose using probiotics as an adjuvant for RFX in upcoming studies. Taken together, we here report the RFX-mediated protection of testicular cells from malathion toxicity. We highlight the cytoprotective role of autophagy in the testicular cells, which degrade dysfunctional mitochondria via mitophagy, thereby combating oxidative stress. An important consideration for the effectiveness of testicular protection is the chronic administration of RFX over an extended period to promote new mitochondria synthesis, hence spermatogenesis.

## Figures and Tables

**Table 1:** Mean testicular biopsy score (MTBS).

Score	Description
1	No cells
2	Sertoli cells without germ cells
3	Only spermatogonia and the epithelium thickness
4	Only a few spermatocytes
5	Many spermatocytes
6	Only a few early spermatids
7	Many early spermatids without differentiation
8	Few late spermatids
9	Many late spermatids
10	Full spermatogenesis

**Table 2:** Body weight of different treatment groups exposed to malathion with or without rifaximin (RFX).

Parameter	Control	RFX	Malathion	Malathion + RFX
Initial body weight *n* = 12	162 ± 8	160 ± 6	165 ± 5	163 ± 7
2 W body weight *n* = 6	194 ± 11	190 ± 9	188 ± 10	189 ± 9
4 W body weight *n* = 6	225 ± 15	222 ± 13	208 ± 17	211 ± 14

## Data Availability

The data that support the finding of this study are available from the corresponding author upon reasonable request.
